# Computational Insights into the Catalytic Mechanism of *Is*‐PETase: An Enzyme Capable of Degrading Poly(ethylene) Terephthalate

**DOI:** 10.1002/chem.202201728

**Published:** 2022-10-25

**Authors:** Eugene Shrimpton‐Phoenix, John B. O. Mitchell, Michael Bühl

**Affiliations:** ^1^ EaStChem School of Chemistry University of St Andrews KY16 9ST St Andrews UK

**Keywords:** catalysis, enzyme, green chemistry, plastics, QM/MM

## Abstract

*Is*‐PETase has become an enzyme of significant interest due to its ability to catalyse the degradation of polyethylene terephthalate (PET) at mesophilic temperatures. We performed hybrid quantum mechanics and molecular mechanics (QM/MM) at the DSD‐PBEP86‐D3/ma‐def2‐TZVP/CHARMM27//rev‐PBE‐D3/dev2‐SVP/CHARMM level to calculate the energy profile for the degradation of a suitable PET model by this enzyme. Very low overall barriers are computed for serine protease‐type hydrolysis steps (as low as 34.1 kJ mol^−1^). Spontaneous deprotonation of the final product, terephthalic acid, with a high computed driving force indicates that product release could be rate limiting.

## Graphical abstract

**The enzyme** ***is*** **‐PETase** degrades poly(ethylene) terephthalate (PET) at mesophilic temperatures. This makes *is*‐PETase a promising candidate for the biodegradation of this common pollutant. The energy profile for the degradation of a PET substrate mimic by *is‐*PETase was calculated by using computational quantum‐mechanical/molecular mechanical methods, and the activation energy for this reaction was calculated to be 34.1 kJ mol^−1^.

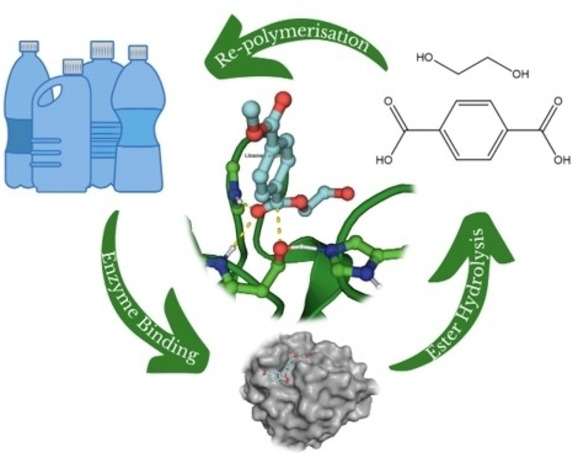

## Introduction

**Figure 1:**
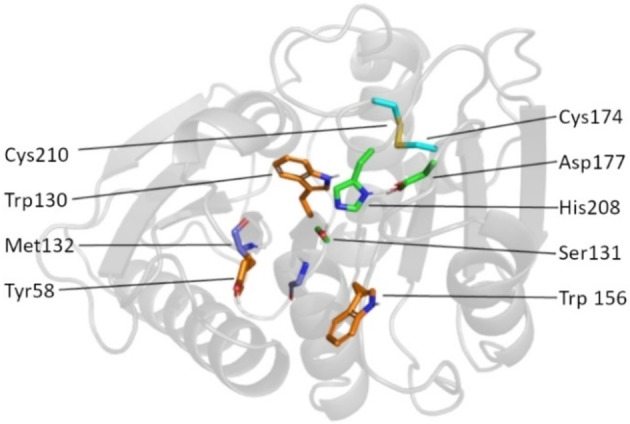
Residues key to the activity of *Is*‐PETase. S131, H208 and D177 form the catalytic triad that acts as the nucleophile in the enzyme's two nucleophilic substitution reactions. The backbone nitrogen atoms of Y58 and M132 act as hydrogen‐bond donors during the reaction; this stabilises negative charges that develop in the tetrahedral intermediates during the nucleophilic substitutions (Figure 2). Aromatic residues Y58, W130 and W156 form π‐stacking interactions with the phenyl rings of PET, making them vital for substrate binding. A disulfide bond between C174 and C210 connects the loops between the α6‐β8 and α4‐β7 domains. These loops contain the residues H208 and D177, respectively.

**Figure 2:**
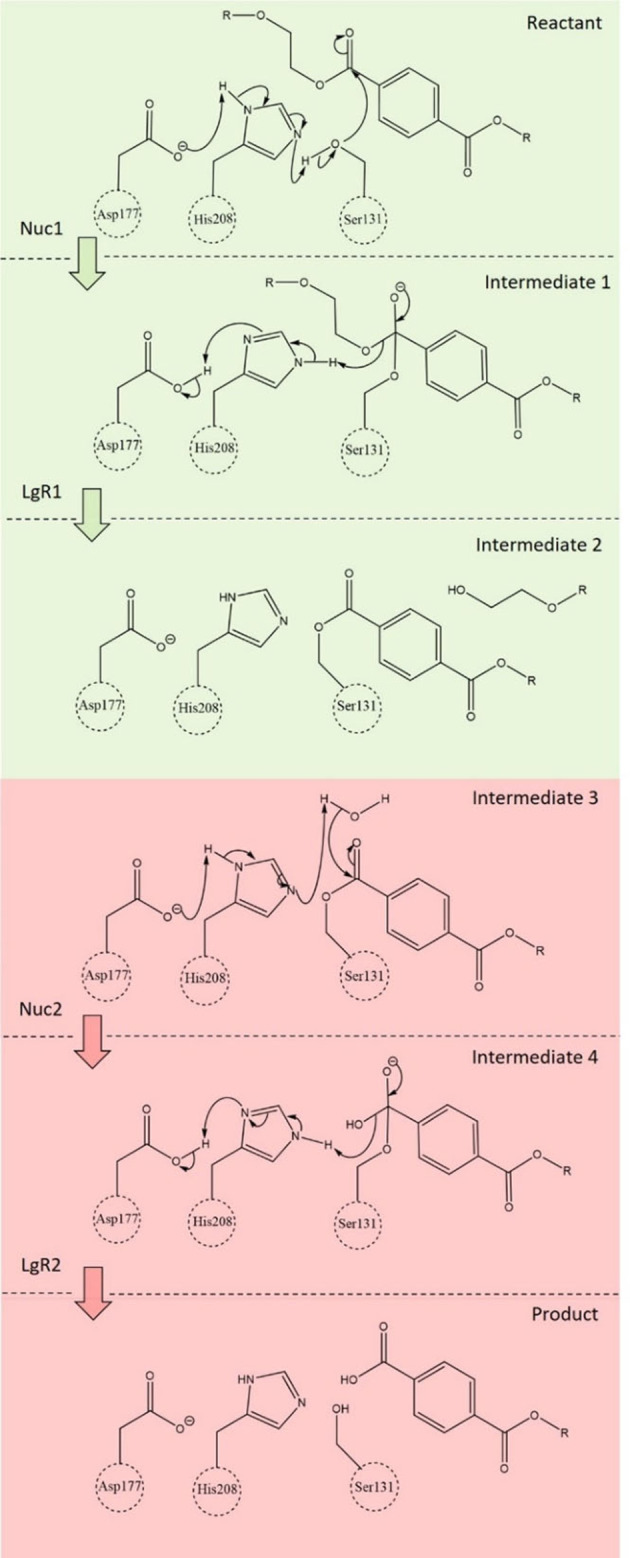
Proposed mechanism for the reaction catalysed by *Is*‐PETase. The reaction has been split into two separate nucleophilic substitution reactions: NS1 (green) and NS2 (red). These reactions each constitute a nucleophilic addition (Nuc1 and Nuc2) and removal of a leaving group (LgR1 and LgR2) step.

In 2016, a culture of bacteria was discovered near a recycling plant outside Sake city in Japan that could degrade the common pollutant PET.^[1]^ The bacterium responsible for this activity was found to be *Idonella sakaiensis*. Using a pair of enzymes PETase and MHETase, this bacterium breaks PET into its monomers ethylene glycol (EG) and terephthalic acid (TA). It was predicted that the organism catabolises TA for a source of carbon and energy. X‐ray crystallography revealed that *Is*‐PETase (EC 3.1.1.101)^[2]^ is a member of the α‐β hydrolase family,^[3]^ while phylogenetic studies showed that *Is*‐PETase belongs to the cutinase family of enzymes. The chemical mechanism, based upon the structure (Figure 1) was proposed to follow two concerted nucleophilic substitution reactions, using a catalytic triad motif conserved in related α‐β hydrolases^[4]^ (Figure 2).

There are several features of *Is*‐PETase that have been shown to be important for the enzyme's ability to degrade PET (Figure 1). The active site is situated in a shallow groove in the enzyme's surface; in comparison to related cutinase enzymes, this groove has been significantly enlarged and contains more aromatic residues.^[5]^ It is thought that these factors assist with substrate binding. The active site is situated upon two loops, a disulfide bridge that is not conserved in related enzymes has been shown to hold these two loops in position, whilst giving the active site flexibility.

Several enzymes had been reported to have PET degradation activity before the discovery of *Is*‐PETase. These enzymes originated from thermophilic bacteria,^[6]^ resulting in high optimum temperatures for enzymatic activity. It has been argued that reaction temperatures in excess of 60 °C are desirable in PETases, as this is the glass transition temperature (*T*_g_) of aqueous PET.^[7]^ For high temperature industrial enzymatic PET degradation, the thermophilic enzyme LCC^[8]^ is the current frontrunner. By contrast, *Is*‐PETase operates most efficiently at mesophilic temperatures, outperforming all other PETases at 30 °C.^[7]^ Mesophilic activity makes *Is*‐PETase a more practical candidate for large‐scale processes, such as waste stream management, as it would be costly to heat such large systems. *Is*‐PETase has potential to be expressed in a wider array of host organisms. *Is*‐PETase has been expressed in green microalgae, it has been suggested that algae will be a better host organism for use of PETases in environmental applications.^[9]^

Several mutagenesis studies have shown that the catalytic activity of *Is*‐PETase can be improved using two techniques. The primary method is the mutation of key residues around the substrate binding site that results in a more hydrophobic binding pocket or enlarges the active site.[^10^, ^11^, ^12^] Improvements in *Is*‐PETase's catalytic efficiency have been achieved through mutations that improve the enzyme's thermostability. A recent study that uses UV absorbance of products to assess the rate of reaction (rather than HPLC) has given us access to the kinetics of *Is*‐PETase's activity. This has revealed that an increase in *Is*‐PETase's thermostability benefits activity by increasing the catalytic rate and by extending the time before the enzyme is deactivated through thermal degradation.^[13]^

QM/MM methods have been used successfully to elucidate the catalytic mechanism of enzymes (see refs. [14]–[19] for selected examples). The activation energies of transition states can be calculated and the highest (rate‐limiting) barrier can then be compared to those measured in vitro. While it is difficult to strictly prove a mechanism this way, the calculations can either furnish support for or rule out the reaction profile in question.

To date, there have been a handful of studies that have applied different QM/MM approaches to explore the catalytic mechanism of *Is*‐PETase. Two of these studies have used the umbrella sampling method to calculate the free energy surface for each reaction step.[^20^, ^21^] Both of these studies concluded that the rate‐limiting step was the initial acylation (Nuc1 in this paper), with calculated free energy barriers of 84.9^[20]^ and 83.7 kJ mol^−1^.^[21]^ Another approach has been to use molecular dynamics to sample the conformational space of the reaction, followed by several independent optimisations of intermediates and transition states using QM/MM methods.^[22]^ By using this approach, the initial acylation and final de‐acylation steps (Nuc1 and LgR2 in this paper) were identified as competing rate limiting steps, with activation energies of 58.2 and 49.8 kJ mol^−1^, respectively.

In this work we propose a different approach to explore the reaction profile (Figure 2) of the degradation of PET by *Is‐PETase*. Our working hypothesis is that a successive catalytic triad mechanism is operating, akin to the standard serine hydrolase mechanism.^[23]^

## Results and Discussion

### Enzyme–ligand complex reproduced by molecular docking

**Figure 3:**
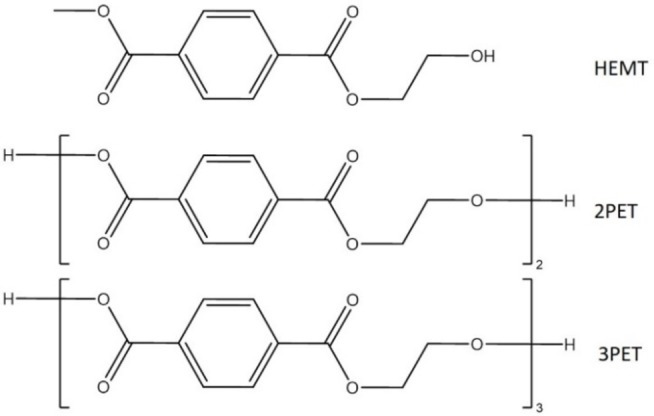
Chemical structures of the ligands docked into *Is*‐PETase.

To generate suitable starting structures for subsequent MD simulations and QM/MM calculations, molecular docking simulations were performed with the enzyme *Is*‐PETase and various lengths of PET chain: 3PET, 2PET and HEMT (Figure 3).

**Figure 4:**
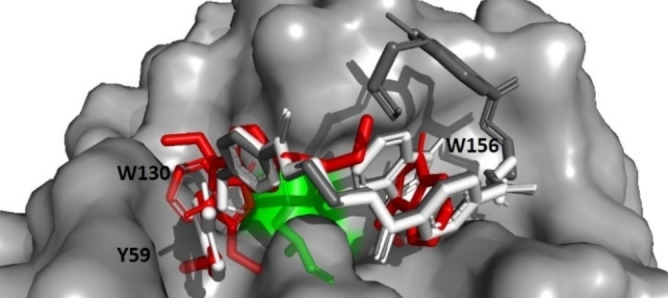
Docking modes of three lengths of PET, alongside the orientations of residues Y59, W130 and W156. HEMT (red), 2PET (white) and 3PET (grey). The catalytic S131 residue is displayed in green.

The aromatic residues Y59, W130 and W156 were set as flexible during these simulations as it has been previously proposed that their mobility (especially the “wobbling” motion of W130) is important during substrate binding.^[11]^ The docking studies (Figure 4) revealed that the portion of the ligands proximal to the catalytic S131 bound in similar modes. The aromatic ring of this moiety forms a π‐stacking interaction with Y59. The docking of 2PET and 3PET revealed the role of W156 as it forms a π‐stacking interaction with the second aromatic ring of each ligand. It can be seen through comparison with the binding mode of HEMT that W156 rotates its side chain to accommodate the longer chains of 2PET and 3PET. The orientations of the catalytic residues S131, D177 and H208 and the reactive ester bond remained consistent across the docking geometries of HEMT, 2PET and 3PET. For this reason and to reduce calculation times, HEMT was chosen as the substrate mimic to be used in the rest of this study.

The binding pose used as a starting point for the subsequent molecular dynamics simulation was selected for its similarity to binding pose found in the PDB structure 5XH3,^[3]^ particularly for the proximity of the ligand's carbonyl oxygen to the backbone nitrogen in the oxyanion hole.

### QM/MM methods used to calculate the energy barriers of the degradation of HEMT by Is‐PETase

Starting with an enzyme–ligand complex generated by molecular docking between Is‐PETase and HEMT, the system was solvated using a series of molecular dynamics simulations (see the Supporting Information). Geometries for the intermediates along the proposed reaction pathway (Figure 2) were generated through a series of restrained and unrestrained QM/MM geometry optimisations. Geometries were generated sequentially (i. e., starting from previously optimised structures) to ensure that geometries remained on the same reaction pathway. The transition state geometries in between these intermediates were then generated using the nudged elastic band (NEB) method. Once the geometries of intermediates and transition states had been found, single‐point calculations were performed to refine their energies. This process was repeated using seven different snapshots from the MD simulation to sample different conformations along the reaction pathway. The resulting reaction pathways are therefore the result of the differing starting geometries obtained from MD simulations.

**Figure 5:**
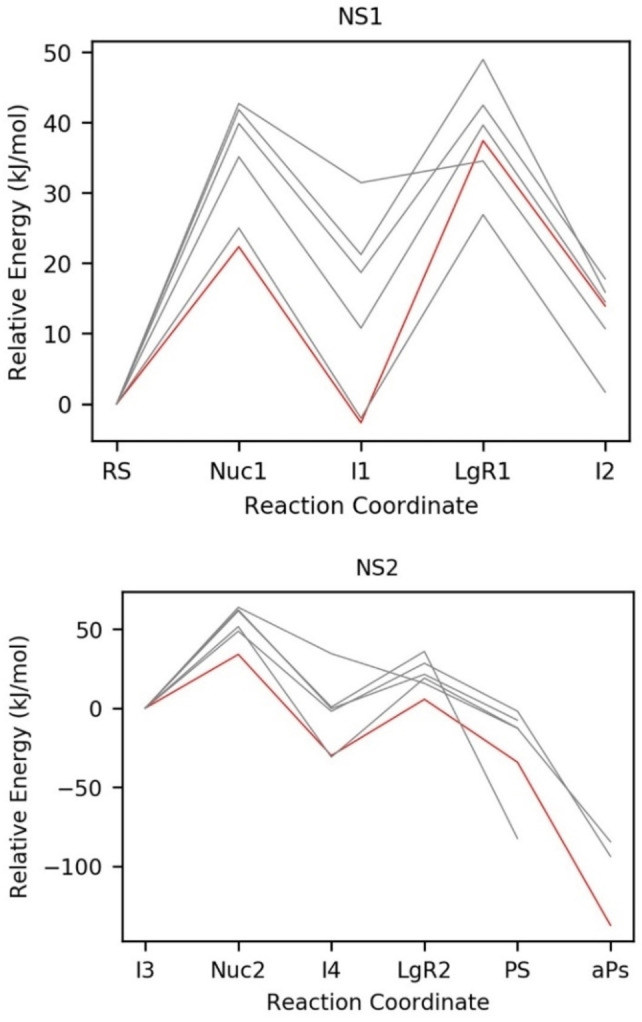
Energy profiles calculated for the two reactions catalysed by *Is*‐PETase (Figure 2), NS1 (top) and NS2 (bottom). The lowest energy profile across each reaction (red) has the rate‐limiting step Nuc2, with an energy barrier of 34.1 kJ mol^−1^. The grey lines represent energy profiles for reactions starting at MD snapshots with higher activation energies.

For the reaction NS1, the reaction pathway (red line in Figure 5, left) with the lowest overall energy barriers had an activation energy of 28.9 kJ mol^−1^ with reaction step LgR1 responsible for the highest energy barrier. For the reaction NS2, the reaction pathway with the lowest overall energy barriers (red line in Figure 5, right) had an activation energy of 34.1 kJ mol^−1^ with reaction step Nuc2 responsible for the highest energy barrier (Figures 2 and 5). Nuc2 has the highest activation energy across reactions NS1 and NS2.

The step connecting intermediates I2 and I3 involves the dissociation of the EG leaving group present in I2, followed by the association of a water molecule which will function as the nucleophile in Nuc2. The driving force for this step could not be calculated with sufficient accuracy using our QM/MM protocol. If the substrate exchange were endergonic (pushing the second profile up relative to the first one) the predicted overall barrier would increase correspondingly. However, seeing that EG and water binding will be dominated by hydrogen‐bonding interactions with similar strength, no significant driving force for this step is to be expected. In addition, it is expected that the dissociation of EG will occur without any significant energy barrier as the molecule forms no strong interactions with either the active site of the enzyme or the bound ligand and no major rearrangement of the protein backbone is required to release the product. The association of a water molecule into a nucleophilic position is similarly expected to occur without a significant energy barrier as H208 will readily form hydrogen bonds with bulk solvent in absence of EG in the active site. We thus assume that the two legs of the reaction in Figure 5 can be seamlessly connected and evaluate our overall barriers assuming zero driving force for the EG/water exchange.

**Figure 6:**
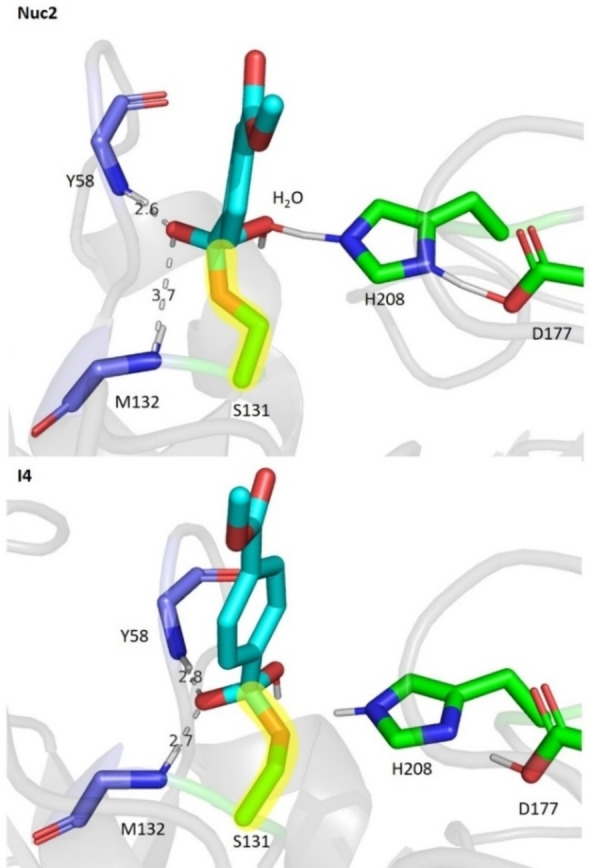
The geometry for the transition state Nuc2 (above) and its subsequent intermediate I4 (below), including key hydrogen‐bond distances in Å. The dihedral angle of the bond from the side chain of S131 to the carbonyl carbon of the substrate (highlighted in yellow) is 168.0° in Nuc2 and 96.6° in I4. These geometries were taken from the reaction pathway of NS2 with the lowest overall activation energy.

The geometry of the Nuc2 transition state shows some stabilisation of the formal negative charge that is forming on the carbonyl oxygen of the substrate en route to the tetrahedral intermediate I4 (through hydrogen bonds involving backbone nitrogen atoms of Y58 and M132, with distances of 2.6 and 3.7 Å, respectively; Figure 6, top). The latter distance is quite long, suggesting less than optimal stabilisation in the TS (this distance closes to 2.7 Å in the subsequent intermediate; Figure 6, bottom). The nucleophilic water molecule must be deprotonated by H208 whilst having an orientation suitable for nucleophilic attack on the carbonyl carbon. To accommodate this, a rotation of the side chain of S131 occurs, pulling the carbonyl oxygen out of the oxyanion hole. (Figure 6). The width of the active site of *Is‐PETase* does not permit the nucleophilic attack of water to occur whilst efficiently stabilising the developing negative charge. Analysis of the transition states Nuc1 and Nuc2 across each reaction pathway calculated revealed that the degree of stabilisation of the negative charge developing on the carbonyl oxygen of the ligand has the largest effect on the activation energy of the corresponding reaction step. The same trend as discussed above holds for the set of Nuc1 and Nuc2 transition state geometries: The shorter the M132 backbone nitrogen – ligand carbonyl oxygen in the transition state, the lower the associated activation energy (*R* ^2^=0.81 for Nuc1 and *R* ^2^=0.87 for Nuc2). Far weaker correlations were found for the equivalent distance with the Y58 nitrogen. This is due to this distance being consistently shorter, with less variance. Plots of this data can be found in the Supporting Information.

This might be an evolutionary compromise that has been made as the expanded active site allows for better substrate binding.

Note that, in particular for step NS1, a notable spread of barriers is computed for the pathways starting from different MD snapshots. However, even the largest overall barrier obtained for all pathways, 62.4 kJ mol^−1^, should be readily surmountable at room temperature.

The barriers for our most favourable pathways are lower than those reported in previous QM/MM studies (between ca. 60–80 kJ mol^−1^ when using M06‐2X and PBE functionals in the QM regions[^20^, ^21^, ^22^]). Our results therefore confirm previous predictions at the lower end of this range and, in view of the spread discussed above, highlight the need to trace multiple pathways in order to avoid spuriously high barriers from a single one.

Several kinetic studies have been performed on *Is*‐PETase using PET films and pellets of various shapes and sizes.[^1^, ^10^, ^13^, ^24^] Because of the potentially different experimental conditions and the uncertainties in our calculated numbers mentioned above, direct comparison of computed and observed kinetic quantities is difficult. It has also been suggested that as the substrate of this reaction is non‐aqueous, classical Michaelis–Menten kinetics cannot be used to relate reaction rates to calculated energy barriers.^[25]^

These energy barriers calculated in this work are suitably low to suggest that none of the hydrolysis steps catalysed by *Is*‐PETase constitute its rate‐limiting step. By a process of elimination, this leaves either substrate binding or product release as candidates for the rate limiting step. This is consistent with the findings from site‐directed mutagenesis studies, where the largest improvements in enzyme activity were obtained through mutations that are thought to affect binding.

**Figure 7:**
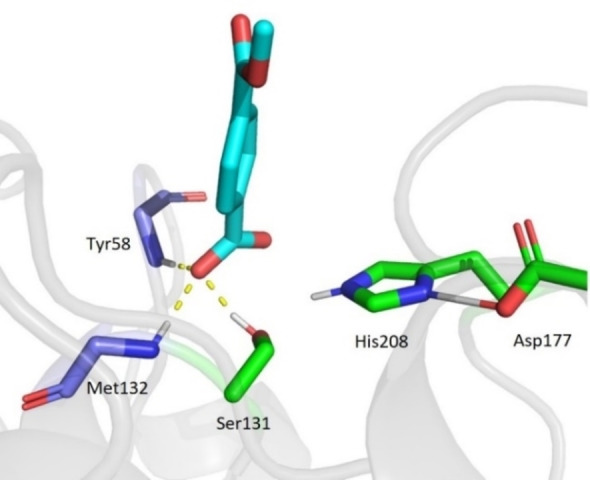
Geometry of an alternative product state. The carboxylic proton has been donated to S131 resulting in a negatively charged product. Hydrogen bonds with the backbone nitrogen atoms of Y58 and M132 as well as the side‐chain oxygen of S131 are shown (yellow dashed lines).

In several calculation steps starting from six MD snapshots, the product geometry (Figure 2) was not found to be stable, instead forming an alternate product state aPS (Figure 7). The carboxylic moiety of the ligand was deprotonated, having transferred to the side‐chain oxygen of S131 causing the ϵ‐nitrogen on H208 and the carboxyl of D177 to be protonated. The energy of this state was calculated to be up to −137.4 kJ mol^−1^ more stable relative to the geometry of intermediate 4 that preceded it (Figure 5). Subsequent MD simulations of the aPS state revealed that the deprotonated product forms strong hydrogen bonds with the active site residues (Figure 6). This caused the ligand to persist in the active site for up to 1 ns. This tendency for the product to deprotonate while inside the active site might thus result in a substantial energy barrier in the product release step of the reaction. This would significantly hinder the enzyme's catalytic efficiency.

## Conclusions

Using QM/MM methods, we have calculated that the lowest energy barrier in the degradation of PET by *Is*‐PETase is between 34.1 and 62.4 kJ mol^−1^, consistent with that of similar esterases. This serves as further support to the hypothesis that *Is*‐PETase operates by using the reaction mechanism described in Figure 2.

The lowest of our computed overall energy barriers is associated with the nucleophilic addition of a water molecule activated by the H208–D177 diad to the enzyme‐bound ligand. Due to this low calculated barrier, we suggest that the overall rate‐limiting step for this reaction is product dissociation. This is further supported by the formation of a stable alternative product, which might inhibit product release.

Given the above results, we recommend modifications to *Is*‐PETase that improve the thermostability of the enzyme. This would allow the enzyme to operate effectively at a higher temperature, which would accelerate the diffusion‐dependent product‐release step.

## Experimental Section

**Molecular docking simulations**: Molecular docking simulations were performed using the AutoDock Vina^[26]^ molecular docking software. Input files were prepared using the AutoDock tools desktop application. Molecular docking was performed to generate geometries for the enzyme‐substrate complex for both the HEMT and HMT ligands.

**Preparation of solvated enzyme through molecular dynamics**: The coordinate structure of the enzyme was obtained from the protein data bank (PDB ID: 5XH3^[3]^). Ordered water and other co‐crystalised molecules were removed in the molecular visualisation program PyMOL.^[27]^ This was necessary to perform subsequent molecular docking simulations.

Molecular dynamics simulations were performed using the program Gromacs^[28]^ to generate an enzyme geometry that is energetically relaxed and solvated. Initially, the enzyme was placed in a cubic solvent box with dimensions of 75 Å. The TIP3P water model^[29]^ was used for solvation. Next several solvent molecules were replaced with seven chloride ions to balance the overall system charge. This resulted in a system with 41545 atoms (3767 protein atoms, 28 ligand atoms, seven chloride ions and 37743 solvent atoms). Disulfide bonds were explicitly modelled between cysteines 174 and 210 and between cysteines 244 and 260. An energy minimisation step was then performed using the steepest descent method with a step size of 0.01 nm and a tolerance of 1000 kJ mol^−1^ nm^−1^. Next an NVT simulation was performed with 50000 steps and a step size of 2 fs, using the V‐rescale thermostat. This was followed by an NPT simulation with 50000 steps and a step size of 2 fs, using the V‐rescale thermostat and the Berendsen barostat. A full production MD simulation was then performed under NPT conditions for a duration of 5 ns using the V‐rescale thermostat and the Parrinello–Rahman barostat. All of the above simulations and minimisations were performed using the CHARMM36^[30]^ forcefield and using periodic boundary conditions. The Verlet cutoff scheme was used for all MD steps, with a 10 Å cutoff distance for electrostatic and Van der Waals interactions. The LINCS algorithm was used to constrain bonds to hydrogen in all MD steps. CHARMM compatible forcefield parameters for the ligands HEMT and HMT were generated using SwissParam.^[31]^ Ligand parameters and mdp input files used are available in the Supporting Information.

**Hybrid QM/MM geometry optimisations**: The intermediate geometries of the enzyme‐substrate complex along the proposed reaction coordinate were generated through a series of hybrid QM/MM geometry optimisations. These were performed using the exchange‐correlation density functional rev‐PBE^[32]^ with the D3(BJ)^[33]^ dispersion correction using the def2‐SVP^[34]^ basis set and the CHARMM27 forcefield^[35]^ In each QM/MM calculation the QM region contained the entire ligand (either HEMT or HMT), atoms in the side chains of residues S131, D177 and H208, and backbone atoms from residues G57, Y58, W130, S131, and M132. Further details of the QM region are included in the Supporting Information. The charge‐shift scheme was used to redistribute charges at the QM/MM boundary. All QM/MM calculations used the electrostatic embedding scheme. Following a geometry optimisation using restraints, an unrestrained geometry optimisation was always used to remove the energy contribution from the restraint. Atoms allowed to move during these calculations were defined as those within a 10 Å distance from the carbonyl carbon of the ligand molecule, this was determined at the start of the first optimisation in each reaction profile, then conserved across subsequent calculations. The functionals used in this work were selected for their high performance against the GMTKN55 benchmark database.^[36]^ QM/MM optimisations were performed in the program ChemShell,^[37]^ using the dl‐find optimiser. The software Orca was used to treat the QM region, while the in‐built dl‐poly method was used to treat the MM region.

**Transition state search through nudged elastic band calculations**: The transition state geometries along the proposed reaction coordinates were generated using the nudged‐elastic band (NEB) method,^[38]^ with an additional climbing image search at the end of each calculation.^[39]^ 12 images were generated by interpolation between the two input structures, then optimised using a spring force constant of 0.01 to ensure the images remain on the reaction profile. The NEB calculations were performed using the rev‐PBE‐D3(BJ) density functional and using the CHARMM27 forcefield.^[40]^ The same atoms allowed to move during QM/MM optimisations were also allowed to move in NEB calculations. NEB calculations were performed in the program ChemShell, using the dl‐find optimiser. The software Orca was used to treat the QM region, while the in‐built dl‐poly method was used to treat the MM region.

**Single point calculations**: The energies of each of the intermediate and transition state geometries were re‐evaluated at a higher computational level. These single‐point energies were calculated using the double‐hybrid density functional DSD‐PBEP86^[41]^ with the D3(BJ) dispersion correction using the ma‐def2‐TZVP basis set^[42]^ and using the CHARMM27 forcefield. QM/MM single point calculations were performed in the program ChemShell. The software Orca was used to treat the QM region, while the in‐built dl‐poly method was used to treat the MM region.

## Conflict of interest

The authors declare no conflict of interest.

1

## Supporting information

As a service to our authors and readers, this journal provides supporting information supplied by the authors. Such materials are peer reviewed and may be re‐organized for online delivery, but are not copy‐edited or typeset. Technical support issues arising from supporting information (other than missing files) should be addressed to the authors.

Supporting Information

## Data Availability

The data that support the findings of this study are openly available in University of St Andrews Research Portal at https://doi.org/10.17630/486acc90‐3be5‐43c2‐aa08‐17c02096bb85, reference number 281306707.
