# Supplementary material for: Computational Insights into the Catalytic Mechanism of Is‐PETase: An Enzyme Capable of Degrading Poly(ethylene) Terephthalate
Source: Chemistry. 2022 Oct 25;28(70):e202201728. doi: 10.1002/chem.202201728 (PMC10091965; doi:10.1002/chem.202201728)
Supplement: Supplementary file 1 — Supporting Information [file CHEM-28-0-s001.pdf]

Chemistry–A European Journal

Supporting Information

Computational Insights into the Catalytic Mechanism of *Is*-PETase: An Enzyme Capable of Degrading Poly(ethylene) Terephthalate

Eugene Shrimpton-Phoenix, John B. O. Mitchell,* and Michael Bühl*

Full scheme for calculations in this project

Provided below is an example of the sequence of calculations used to generate the geometries of intermediates and transition states along the proposed reaction pathway for *Is*-PETase. Once these geometries were generated, single-point calculations were performed. Geometries for reactions NS1 and NS2 were generated separately. For reaction NS2 the geometry for the product was generated first, with intermediate 4 and intermediate 3 produced working backwards with respect to the reaction coordinate. The workflow sketched in Scheme S1 below was applied in calculations for Wild-Type *Is*-PETase and S163C-S194C *Is*-PETase with HEMT as the substrate as well as Wild-Type *Is*-PETase with HEMF as the substrate. In the Scheme S1, each calculation uses the output of the previous calculation as its input.

All molecular docking simulations were performed using Autodock Vina. All Molecular Dynamics simulations were performed using Gromacs with the CHARMM36 forcefield and custom ligand parameters generated using SwissParam. All QM/MM calculations were performed in the software Chemshell, with program ORCA used to treat the QM region and the in-built DL-Poly protocol used to treat the MM region. For all QM/MM geometry optimisations and Nudged-Elastic-Band calculations the QM region was treated at the rev2PBE-D3(BJ)/def2-SVP level while the MM region was treated using the CHARMM27 forcefield with custom ligand parameters generated using SwissParam. For all QM/MM single-point energy calculations the QM region was treated at the DSD-PBEP68-D3(BJ)/ma-def2-TZVP level, while the MM region was treated using the CHARMM27 forcefield with custom ligand parameters generated using SwissParam.

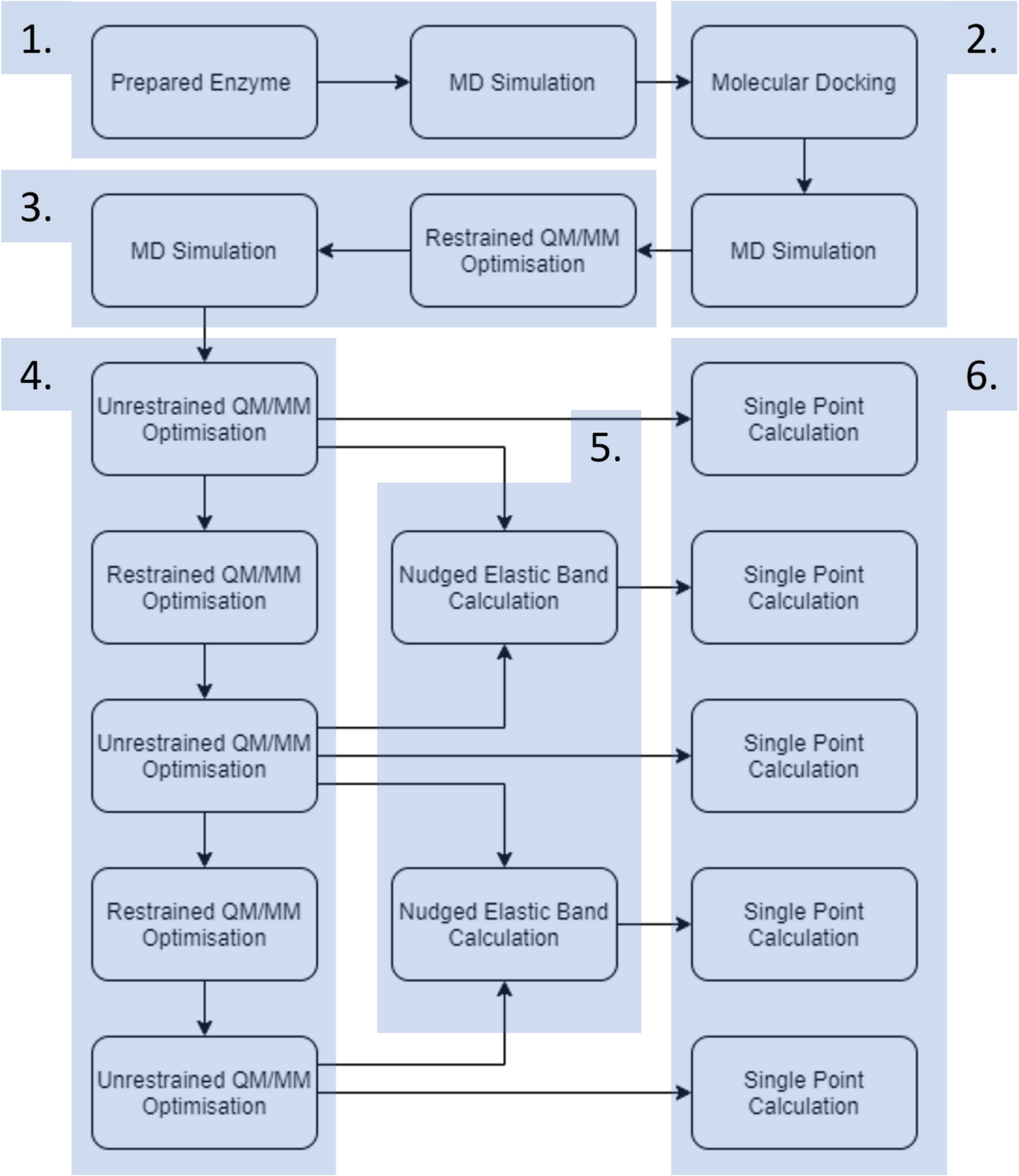

Scheme S1: Workflow of calculations performed in this work.

In detail, the following steps were involved:

1. Starting with an enzyme PDB file that has been cleaned, a MD simulation is performed using the CHARMM36 forcefield with some bespoke parameters as specified below. This is to obtain an enzyme structure equilibrated in the MD forcefield. Prior to molecular docking, the solvent and chloride counter-ions are removed from the enzyme file.
2. Molecular docking is performed to obtain an enzyme-ligand complex. For NS1 the ligand HEMT of HEMF is used; for NS2 the ligand HMT or HMF is used. The enzyme-ligand complex is re-solvated using a MD simulation.
3. Starting from a snapshot of the previous MD simulation at 100 ps, a restrained QM/MM optimisation is performed. Two restraints of 2.9 Å are applied between each of the backbone nitrogen atoms of Y58 and M132 and the reactive carbonyl oxygen of the ligand.

This creates large solvent cavities. To remove these cavities, water molecules and ions are removed then reintroduced. This is followed by a MD simulation with the coordinates of the enzyme and ligand frozen.

4. An unrestrained QM/MM calculation is performed to generate the reactant geometry in NS1 and product geometry in NS2. A restrained QM/MM optimisation is then performed with a restraint of 1.3 Å between the hydroxyl oxygen of S131 and the carbonyl carbon of the ligand, the geometry produced is then optimised in an unrestrained QM/MM calculation to generate the geometry of intermediate 1 in NS1 and intermediate 4 in NS2. A further restrained QM/MM optimisation is then performed with a restraint of 1 Å between the hydroxyl hydrogen (which by this point is bound to H208) of S131 and the leaving group of the ligand (in NS1 this will be an ethylene glycol leaving group, in NS2 this will be a water leaving group). An unrestrained QM/MM calculation is then performed to generate a geometry for intermediate 2 in NS1 and intermediate 3 in NS2.
5. Nudged Elastic Band calculations are used to generate geometries of transition states in between intermediates sequential along the reaction profile.
6. Single point calculations are performed using geometries of intermediates and transition states to create an energy profile for the reaction.

Full Single point calculation results

The single-point energies of each intermediate along each calculated reaction pathways are collected in Tables S1 and S2 below

Snapshot: Step	100	150	300	450	600	750	900
**RS**	0	0	0	0	0	0	0
**NUC1**	22.3	25.0	42.7	39.8	41.8	35.1	45.5
**I1**	-2.7	-2.0	31.4	18.7	21.2	10.9	38.4
**LGR1**	37.4	26.9	34.5	42.5	48.9	39.6	62.4
**I2**	13.9	1.7	10.7	17.8	15.9	14.5	25.3

Table S1: Relative energies of minima and TSs or the first nucleophilic substitution NS1 (in kJ mol^{-1} relative to the energy of the corresponding RS geometry)

Snapshot: Step	100	150	300	450	600	750
**I3**	0	0	0	0	0	0
**NUC2**	34.0	48.7	51.6	63.8	61.5	62.2
**I4**	-30.0	-2.0	-30.9	34.5	0.7	-0.3
**LGR2**	5.6	28.4	19.0	15.7	35.9	21.4
**PS**	-34.2	-1.9	-12.6	-12.7	-82.4	-7.6
**APS**	-137.4	-93.8	n.a	-84.5	n.a.	n.a.

Table S2: Relative energies of minima and TSs or the second nucleophilic substitution NS2 (in kJ mol^{-1} relative to the energy of the corresponding I3 geometry). PS refers to the geometry of the product while APS refers to the geometry where the product has deprotonated the side-chain of Ser131.

This data is displayed in a box and whisker plots below:

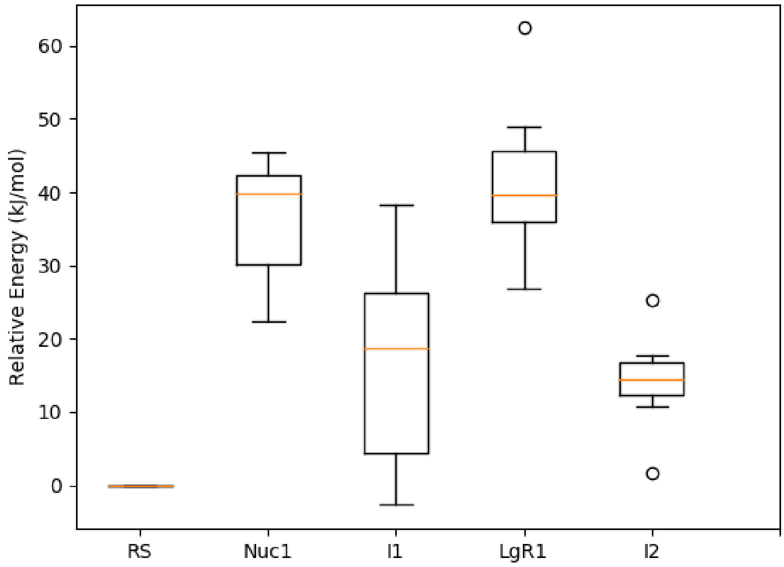

Figure S1: Box and whisker plot of the calculated relative energies of the intermediates and transition states in the reaction NS1. The broadest spread in energies can be observed for I1 geometries, this is most likely due to varying degrees of stabilisation of the negative charge situated upon the ligand's carbonyl oxygen. The standard deviations in relative energies for geometries RS, Nuc1, I1, LgR1 and I2 are 0.0, 9.0, 15.7, 11.4 and 7.1 kJ mol^{-1} respectively.

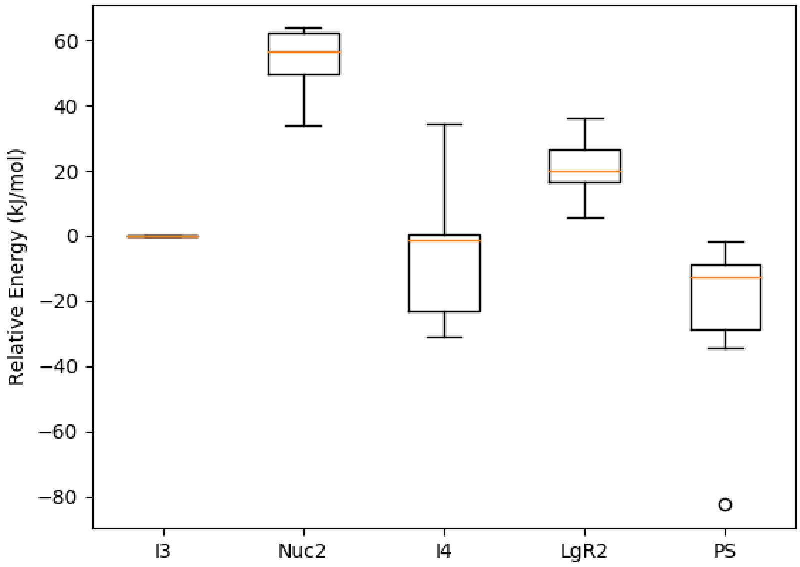

Figure S2: Box and whisker plot of the calculated relative energies of the intermediates and transition states in the reaction NS2. The broadest spread in energies can be observed for I4 geometries, this is most likely due to varying degrees of stabilisation of the negative charge situated upon the ligand's carbonyl oxygen. The standard deviations in relative energies for geometries I3, Nuc2, I4, LgR2 and PS are 0.0, 11.4, 24.2, 10.5 and 30.1 kJ mol^{-1} respectively.

It is worth noting that the variance in our data is largely caused by differences in geometries due to different starting points along the molecular dynamics trajectory.

Non-standard MD parameters for ligands

The following ligands were parameterised using the web service SwissParam[1]: HEMT, HMT,HEMF and HMF. These parameters were used in conjunction with standard CHARMM36 in our MD simulations. In our QM/MM calculations the entirety of the ligand was included in the QM-region. As a result, only the non-bonding terms from these non-standard parameters were used in the QM/MM calculations. These parameters are available for download in the ESI of this paper.

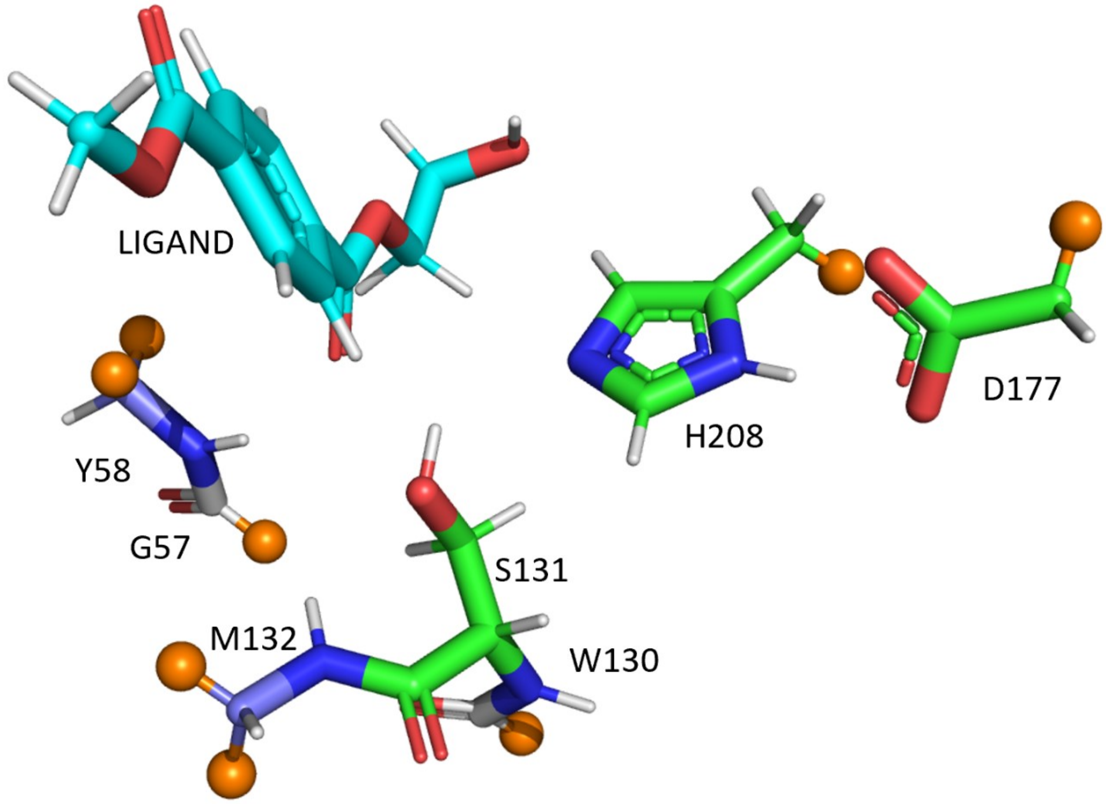

Figure S3: A representation of a sample QM region. The link atoms are displayed as orange spheres. In this case, the LIGAND is HEMT. In calculations relating to reaction NS2, the LIGAND HMT is used instead.

Selection of QM region

The QM region was selected such that only carbon-carbon bonds were cut. Care was also taken to preserve CHARMM groups. The following QM region was used for all QM/MM calculations:

G57 (C O), Y58 (H HN CA HA), W130 (C O), S131 (all atoms), M132 (N HN CA HA), D177 (all atoms in sidechain), H208 (all atoms in sidechain), LIGAND (all atoms). See Figure S1 for a visual representation.

PDB of equilibrated and solvated system

An example .pdb file for *Is*-PETase within an equilibrated solvent system is available to download in the ESI of this paper.

PDB files of QM/MM optimised structures

An example geometry for each intermediate and transition state is available to download in the ESI of this paper as a .pdb file.

Additional comments on product release

For pathways in which the product did not spontaneously deprotonate, the product was deprotonated manually. This was done by applying a QM/MM optimisation with a restraint applied between the carboxyl proton of the product and the hydroxyl oxygen of S131, with a length of 1 Å. A further QM/MM optimisation was then performed without any restraints. In each case, the deprotonated form of the product was significantly more stable than the product state with an average energy difference of -121.2 kJ mol^{-1}, this represents a far larger energy difference than the largest energy barrier calculated in this work.

Details for Docking Simulations

Molecular docking simulations were performed using the AutoDock Vina program. The search area for the docking was defined by a grid box. This grid box was centred upon the γ-oxygen of the catalytic residue Ser131. The grid box has a size of 40 Å by 20 Å by 30 Å in the x, y and z dimensions respectively. This minimised the search area whilst fully enclosing the enzyme's active site. For all docking simulations, an exhaustiveness parameter of 16 was used. The 8 binding modes with the highest calculated affinities were selected as outputs from each molecular docking simulation. As a test, the values for exhaustiveness and number of output binding modes were increased: altering either parameter yielded no significant change in the resultant binding modes.

Binding modes were selected for their similarity to the binding mode of HEMT found in PDB: 5xh3. Features of this binding mode included proximity of the ligand's reactive carbonyl oxygen to the backbone nitrogen of Tyr58 and Met132. Additionally the proximity of the ligand's reactive carbonyl carbon to the γ-oxygen of the catalytic Ser131 was considered important.

Selection of active region for QM/MM calculations

During each QM/MM optimisation or Nudged-Elastic-Band calculation, only atoms in a predefined active region are allowed to move. The atoms in this region were defined prior to the first optimisation calculation performed for each reaction pathway. All atoms in the protein and ligand were included in the active region. Using the starting geometry from the molecular dynamics snapshot, atoms in solvent molecules with any atoms within 10 Å of the reactive carbonyl carbon of the ligand were also included in the active region. For each reaction pathway, once the atoms in the active region was defined, this atom list remained consistent.

Plots of oxyanion hole distances

Presented here are the hydrogen bonding distances of the ligand's carbonyl oxygen and the nitrogen atoms in the oxyanion hole (the backbone nitrogen atoms of Tyr58 and Met132), for transition state geometries Nuc1 and Nuc2. The distance M132N to carbonyl oxygen (OAH M132) was found to correlate strongly with the activation energy of the associated nucleophilic attack, while the Y58N to carbonyl oxygen (OAH Y58) distance was found to have little correlation with the steps activation energy. As the OAH M132 distance was found to be consistently longer than the OAH Y58 distance and is often longer than the standard hydrogen bond (2.6 - 3.3 Å) this trend can be rationalised by the following: reduction in the OAH M132 distance increases the strength of the hydrogen bonding interaction, which increases the stabilisation effect on the developing negative charge in both Nuc1 and Nuc2 transition states. This lowers the energy of the transition state and thus reduces the activation energy of the step. A weak correlation is found for OAH Y58 due as this distance consistently falls within the range of standard hydrogen bonds, with little variation.

Snapshot	OAH Y58	OAH M132	Activation Energy
100	2.8	3.0	22.3
150	2.7	3.3	25.0
300	2.7	3.7	42.7
450	2.8	3.4	39.8
600	2.7	3.5	41.8
750	2.8	3.4	35.1
900	2.7	3.7	45.5

Table S3: For each Nuc1 transition state geometry: the oxyanion hole distances and the reaction step's associated activation energy.

Snapshot	OAH Y58	OAH M132	Activation Energy
100	2.6	3.1	34.1
150	2.6	3.2	48.7
300	2.6	3.4	51.6
450	2.6	3.6	63.8
600	2.6	3.6	61.5
750	2.6	3.4	62.2

Table S4: For each Nuc2 transition state geometry: the oxyanion hole distances and the reaction step's associated activation energy.

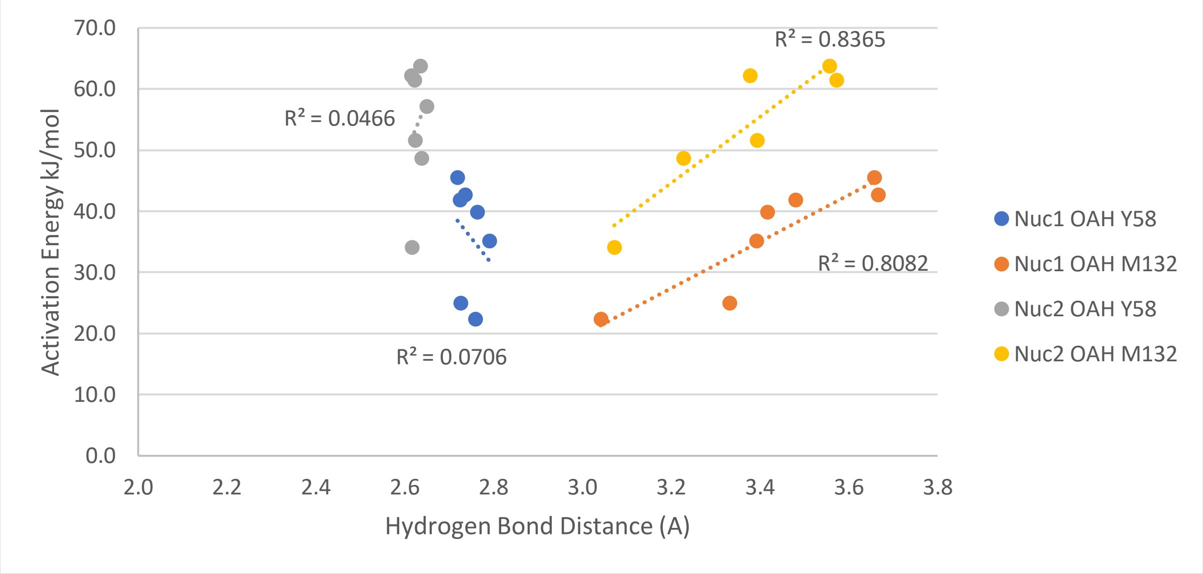

Figure S4: Plot of oxyanion hole hydrogen bonding distances against activation energies for transition state geometries Nuc1 and Nuc2.
